# A systematic review and meta-analysis on incidence of prostate cancer in Iran

**DOI:** 10.15171/hpp.2019.13

**Published:** 2019-05-25

**Authors:** Asaad Moradi, Mohammad Zamani, Emadoddin Moudi

**Affiliations:** ^1^Department of Urology, Firozgar Hospital, Iran University of Medical Sciences, Tehran, Iran; ^2^Student Research Committee, School of Medicine, Babol University of Medical Sciences, Babol, Iran; ^3^Cancer Research Center, Health Research Institute, Babol University of Medical Sciences, Babol, Iran; ^4^Clinical Research Development Center, Shahid Beheshti Hospital, Babol University of Medical Sciences, Babol, Iran; ^5^Department of Urology, Babol University of Medical Sciences, Babol, Iran

**Keywords:** Prostate neoplasms, Incidence, Epidemiology, Systematic review, Meta-analysis

## Abstract

**Background:** Prostate cancer is a global health concern. In Iran, its epidemiology is not precisely recognized. We aimed to evaluate incidence of prostate cancer among Iranian populations.

**Methods:** In this systematic review, we searched the databases PubMed, Web of Sciences, Scopus and Google Scholar for English studies and the databases Magiran, Scientific information Database, IranMedex and IranDoc for Persian studies, using related keywords. The cross sectional articles published from inception to 31 December 2018 were included. Meta-analysis was conducted on the collected data with STATA software using random effects model.

**Results:** Out of 763 articles initially obtained, 9 articles were finally included after applying the predefined exclusion criteria. Analysis of 9 studies on the incidence of prostate cancer showed a crude rate of 7.1 per 100000 population (95% confidence interval [CI]: 5.6-8.6). Also, the pooled age-standardized incidence rate was 8.7 per 100000 (95% CI: 6.7-10.4). Studies performed in the period 2004-2012 had significantly a higher pooled estimate of the crude incidence rate (9.2 per 100000 [95% CI: 7.9-10.4]) compared with those conducted in the period 1996-2003 (4.5 per 100000 [95% CI: 2.8-6.2]). This trend was also observed based on the age-standardized incidence rate (11 per 100000 [95% CI: 9.4-12.5] versus 6.3 per 100000[95% CI: 4-8.5]).

**Conclusion:** Despite low rate of prostate cancer occurrence in Iran, it is recommended that preventive measures be taken against this disease by health policymakers. Also, more epidemiological studies are needed to better find out the pattern of prostate cancer among Iranian populations.

## Background

Prostate cancer is presently one of the major health concerns in the world.^1^ It is globally the second most frequent malignancy and the fifth leading cause of cancer deaths for males.^2,3^ Its epidemiology varies between different regions, that is, western countries has lower rates than eastern countries.^4^

According to the Global Burden of Disease study, 1.4 million new cases of prostate cancer was recorded in 2016 and it was determined as the most common incident cancer in males. Also, 381 000 deaths were attributed to this disease.^5^ It is expected that new cases of prostate cancer and the death number will reach to 1.7 million and 499 000 people around the world by 2030, respectively.^6^

Iran, a developing country, is experiencing an epidemiological transition. The burden of cancer has been associated with an ascending trend in this country over the past decades.^7,8^ According to the report by Ministry of Health in Iran, cancer is the third cause of death and prostate cancer is one of the ten most frequent cancers.^9^ A previous study in 2005 revealed that it is the second most prevalent cancer among genitourinary cancers in Iranian males.^10^ So, strategic planning is needed to better control this disease in Iran. To accomplish this aim, determining epidemiological patterns of the cancer is necessary. Cancer registries established across Iran have been a positive step in this regard. However, no inclusive and precise report is available on the epidemiology of prostate cancer in this country. For this reason, we systematically reviewed the literature on the incidence of prostate cancer in Iran and attempted to provide comprehensive information about this subject. These data would be helpful to health policy-makers and physicians for better planning and prevention and also making decisions on management and treatment of the patients with prostate cancer.

## Materials and Methods

### Information sources and search strategy

The authors undertook a literature search of the following databases from inception to 31 December 2018: PubMed, Web of Sciences, Scopus and Google Scholar. The related terms were searched in the Medical Subject Headings (MeSH) database, and finally, the keywords “prostatic neoplasm” OR “prostatic neoplasms” OR “prostate cancer” OR “prostate cancers” OR “cancer of prostate” OR “cancer of the prostate” AND “epidemiology” OR “prevalence” OR “incidence” OR “frequency” AND “Iran” OR “Iranian” were selected. The search was limited to Title/Abstract. It was also limited to the affiliation “Iran”. The Persian equivalents of the keywords were used for searching the national Persian databases, including Magiran and Scientific Information Database (SID), IranMedex and IranDoc. We also manually searched the reference lists of each included article for additional sources.

The present systematic review and meta-analysis was conducted according to the PRISMA (Preferred reporting items for systematic review and meta-analysis) guideline.^11^ The protocol of this systematic review and meta-analysis has been registered in the PROSPERO registry (CRD42018102421).

### Inclusion and exclusion criteria

We included all English and Persian cross-sectional studies that reported the incidence of prostate cancer among Iranian population. The exclusion criteria for our study were the following: Reviews, case reports, correspondences and editorials, Duplicate articles or studies reporting on the same population, Case-control studies, Studies included subjects with other specific conditions (e.g., cancer of other organs, renal transplantation), and Articles without explicit methodology or results.

### Study selection and data extraction

Two authors (MZ and EM) independently assessed the study eligibility by reviewing the titles and abstracts of all potential citations. Discrepancies were resolved by consensus between the reviewers. Full-text of the relevant articles was assessed. The following data were extracted from each included study: first author’s name, location of study, study period, population size, diagnostic method, and crude rate and/or age-standardized rate (ASR) of the incidence. If multiple publications of the same population were retrieved, the one giving more detail was chosen.

### Statistical analysis

The meta-analysis was conducted using the statistical software STATA V13 (StataCorp, College Station, TX, USA). Point estimates and their 95% confidence intervals (CIs) were calculated. When a study reported the incidence rate in different times, we included them as a separate report in the analysis, if applicable. We performed a subgroup analysis according to the region for incidence of prostate cancer. Also, a sub-analysis was conducted to evaluate the incidence stratified by time. In this regard, we split the study period into 1996-2004 and 2005-2012. Heterogeneity was checked using the I^2^ index. We used random effects model to combine the effect estimates from studies. The cumulative meta-analysis was conducted based on year of publication. Publication bias was assessed using a funnel plot, Begg’s and Egger’s tests.

## Results

**Figure 1:**
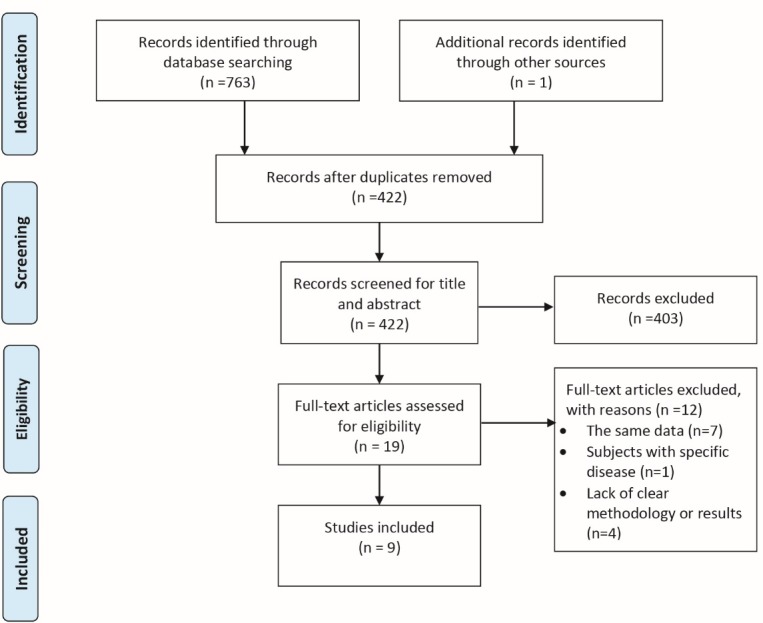
Flow diagram of study selection.

The search yielded a total 763 articles: 562 from the English databases and 201 from the Persian databases. After assessing title and abstract, 403 studies were excluded due to meeting the predefined exclusion criteria. Then, full-text of the remaining 19 articles was retrieved for more detailed evaluation. The manual search found 1 additional article. Finally, 9 papers were included in the study. Figure 1 shows the results of search strategy according to the PRISMA (Preferred reporting items for systematic review and meta-analysis) flow diagram.^11^

### Incidence of Prostate Cancer

**Figure 2:**
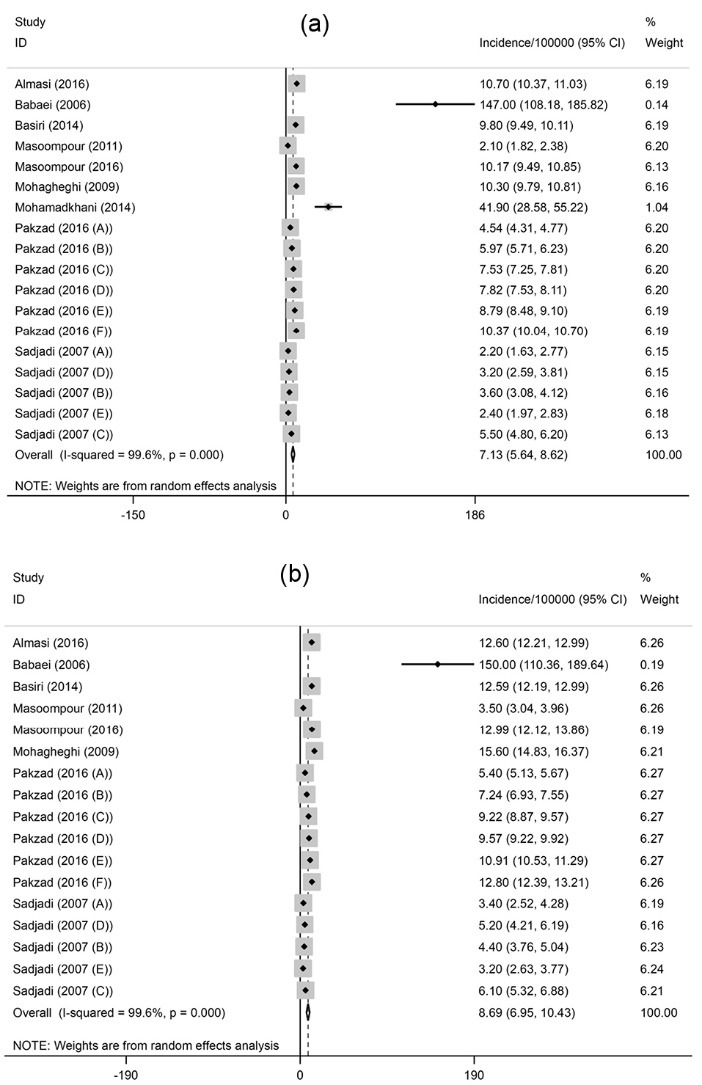
(a) Forest plot of the pooled crude incidence of prostate cancer in Iran. (b) Forest plot of the pooled age-standardized incidence of prostate cancer in Iran.

A total of 9 articles on the incidence were finally included (Table 1). Three studies reported the incidence rate of prostate cancer in national populations and others were restricted to the local cancer registries, including Ardabil, Fars, Golestan, Guilan, Kerman, Mazandaran, Semnan and Tehran. The studies period ranged from 1996 to 2012. All of the methods used for diagnosis of prostate cancer was histopathology. The overall pooled crude incidence rate of the cancer was 7.1 per 100000 population (95% CI: 5.6-8.6) (Figure 2a). Heterogeneity existed between estimates (I^2^=99.6%; *P* < 0.001). In addition, the ASR was estimated to be 8.7 per 100 000 (95% CI: 6.7-10.4; I^2^=99.6%; *P* < 0.001) (Figure 2b).

**Figure 3:**
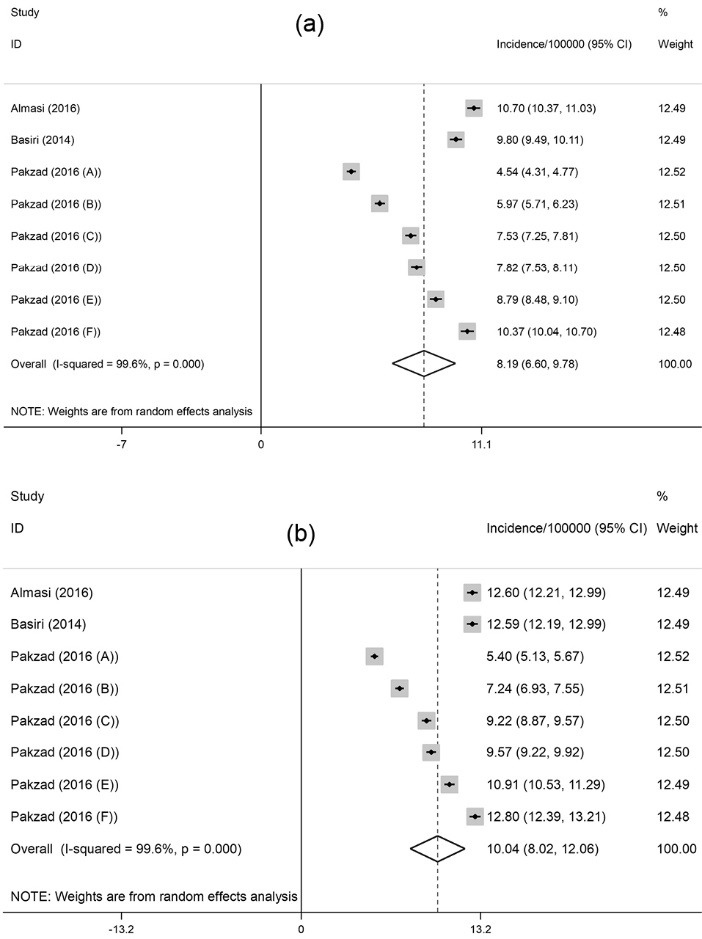
(a) Forest plot of the pooled crude incidence of prostate cancer among national population in Iran. (b) Forest plot of the pooled age-standardized incidence of prostate cancer among national population in Iran.

An analysis was also conducted on the nation-wide reports to estimate the incidence rate among national population. As exhibited in Figure 3a, the pooled crude incidence rate was 8.2 per 100 000 (95% CI: 6.6-9.8; I^2^=99.6%; *P* < 0.001). Besides, the pooled ASR incidence was 10 per 100 000 (95% CI: 8-12.1; I^2^=99.6%; *P* < 0.001) (Figure 3b).

**Figure 4:**
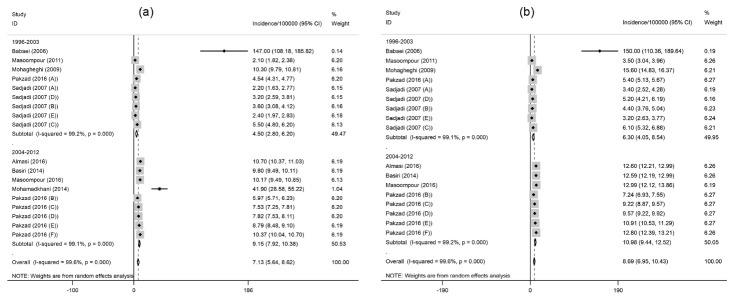
(a) Forest plot of the pooled crude incidence of prostate cancer by study period. (b) Forest plot of the pooled age-standardized incidence of prostate cancer by study period.

The results of subgroup analysis indicated that the crude incidence rate of prostate cancer is significantly higher in the period 2004-2012 (9.2 per 100 000 population [95% CI: 7.9-10.4; I^2^=99.2%; *P* < 0.001]) in comparison with 1996-2003 (4.5 per 100 000 population [95% CI: 2.8-6.2; I^2^=99.1%; *P* < 0.001]) (Figure 4a). This increasing trend was also found for the ASR incidence (11 per 100 000 [95% CI: 9.4-12.5; I^2^=99.2%; *P* <0.001] in the period 2004-2012 versus 6.3 per 100000 in the period 1996-2003 [95% CI: 4-8.5; I^2^=99.1%; *P*<0.001]) (Figure 4b).

**Figure 5:**
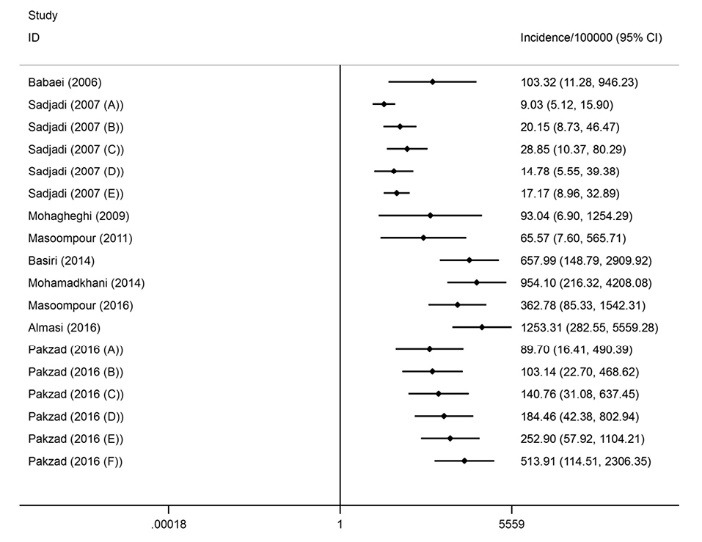
Result of cumulative meta-analysis based on year of publication.

A cumulative meta-analysis of the included studies was performed based on publication year. Figure 5 exhibits the cumulative meta-analysis.

**Figure 6:**
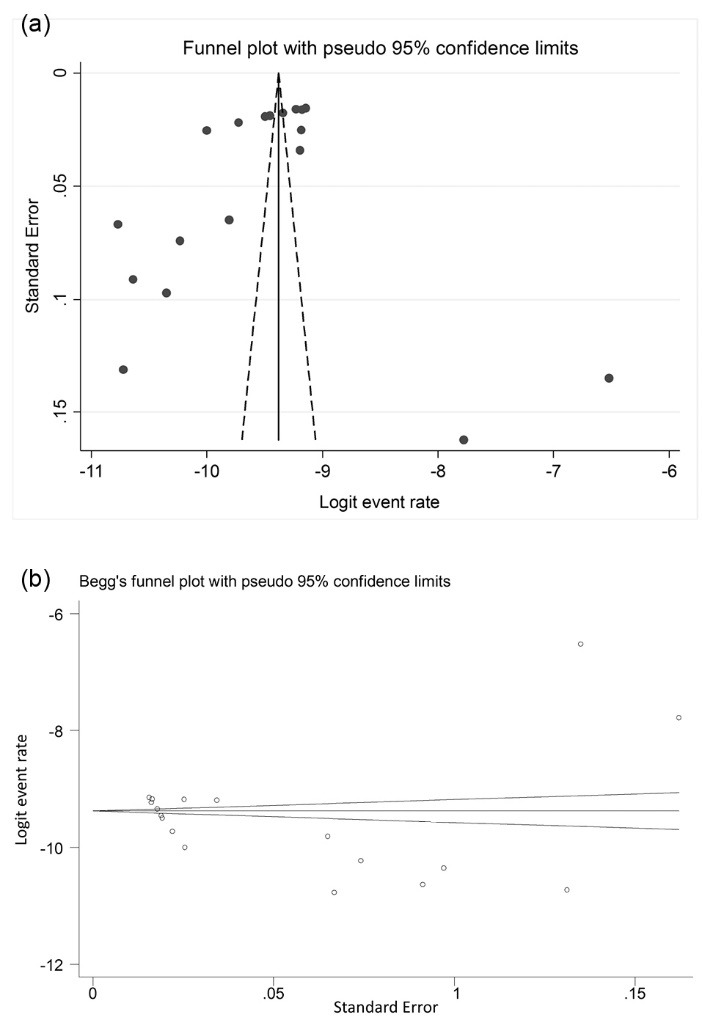
(a) Funnel plot to assess publication bias across the studies. (b) The Begg’s test for publication bias.

Visual inspection of the funnel plot did not indicate any potential publication bias (Figure 6a). Begg’s and Egger’s tests also showed no significant evidence of publication bias among the included studies (Begg’s test, *P* = 0.161; Egger’s test, *P* = 0.204). Figures 6b and 6c are related to Begg’s and Egger’s tests, respectively.

## Discussion

This review presented pooled epidemiological data of prostate cancer in Iran. The overall ASR incidence of the prostate cancer was estimated to be 8.7 per 100000 population, and this rate was 10 per 100000 population based on nation-wide reports. Our figure is near to the report by GLOBOCAN in 2018, with an estimated rate of 16.6 per 100000.^20^ Distribution of prostate cancer regionally changes worldwide. In 2018, its incidence was highest in Northern America, Europe and Oceania with ASR of 130, 125.1 and 113.8 per 100000, respectively, and the lowest in Asia and Africa with ASR of 12.8 and 12.6 per 100000, respectively.^20^ These variations can also be observed at lower scales between the neighboring countries with a close proximity. In comparison to Iran, prostate cancer was reported to have a ASR of 4.5 per 100000 in Afghanistan versus 41.7 per 100000 in Turkey.^20^ Recently, a meta-analysis was published on incidence of prostate cancer in Iran and reported an ASR of 9.11 per 100000,^21^ however, our article has superiorities over that. Firstly, we presented both of pooled crude and ASR incidence of prostate cancer in our study. Secondly, we tried to exclude the duplicates to prevent overestimation of the values. Thirdly, we did a sub-group analysis by study period to assess the trend of prostate cancer incidence.

Altogether, it is known that occurrence of prostate cancer is considerably more in developed countries than in developing countries. This difference can be mainly due to population-based prostate cancer screening and use of advanced diagnostic methods (PSA testing and TURP) which are expanding in most developed countries, whereas they have not been widely taken into consideration in developing communities.^22-24^ Hence, it is predictable that number of new cases will increase by earlier detection of the cancer in western nations more than in the eastern nations.

As determined, incidence of prostate cancer in Iran had a higher rate in the period 2004-2012 in comparison with 1996-2003, which is in consistent with the global increasing trend.^4,25^ This increase in the incidence can not only result from success of screening program for prostate cancer in Iran, but also from aging population and behavioral factors, such as smoking and westernized diets.^7,26,27^

Genetic factors have been discussed to be linked to the prostate cancer in some articles on Iranian population. For instance, genotypes of rs4977574, rs1333048 and rs10757278 from INK4 locus, and rs12826786 T allele from HOX transcript antisense RNA, were recently reported.^28,29^ A case-control study in west of Iran also showed that western unhealthy dietary pattern was associated with increased risk of prostate cancer (OR: 3.4), while a healthy dietary pattern was associated with decreased risk of prostate cancer (OR: 0.24).^30^

A limitation for our study was the significant heterogeneity seen for the pooled analyses. In relation to the incidence, we witnessed high heterogeneity even after analysis of the data on national populations (excluding the reports at local scales). Altogether, a number of factors could be considered as sources of heterogeneity, such as variations in population size and age distributions, study date, and diagnostic criteria discussed before. High heterogeneity is usually seen in systematic reviews and meta-analyses on epidemiological surveys and it is not unexpected.^31^ Lack of clear information in four studies cause excluding them, while their data might be useful to the analyses. A good point about our review was lack of publication bias among individual studies, suggesting that our results are reliable.

## Ethical approval

Not Applicable.

## Competing interests

The authors declare that they have no competing interests.

## Funding

None.

## Authors’ contributions

Study design: EM, AM; Data collection: EM, MZ; Data analysis: MZ; Drafting the manuscript: EM, MZ; Intellectual input & manuscript revision: AM; All authors have read and approved the final manuscript. They also accepted responsibility for all aspects of the work.

**Table 1:** Studies evaluating the incidence of prostate cancer in Iran

**Location**	**Study date**	**Study population**	**Confirmation of diagnosis**	**Diagnostic criteria**	**Crude rate (per 100000)**	**ASR (per 100000)**	**Author**
Ardabil	1996-2000	2636364	Histopathology	ICD-O-3	2.2	3.4	Sadjadi^12^ (A)
Fars	1998-2002	10666667	Histopathology	ICD-O	2.1	3.5	Masoompour^13^
Fars	2007-2010	8397247	Histopathology	ICD-O	10.17	12.99	Masoompour^14^
Golestan	1996-2000	3312500	Histopathology	ICD-O-3	3.2	5.2	Sadjadi^12^ (D)
Guilan	1996-2000	5055556	Histopathology	ICD-O-3	3.6	4.4	Sadjadi^12^ (B)
Kerman	1996-2000	5000000	Histopathology	ICD-O-3	2.4	3.2	Sadjadi^12^ (E)
Kerman	2010	90692	Histopathology	NA	41.9	NA	Mohamadkhani^15^
Mazandaran	1996-2000	4309091	Histopathology	ICD-O-3	5.5	6.1	Sadjadi^12^ (C)
Nation-wide	2003	34096916	Histopathology	ICD-O C61	4.54	5.4	Pakzad^16^ (A)
Nation-wide	2004	34706868	Histopathology	ICD-O C61	5.97	7.24	Pakzad^16^ (B)
Nation-wide	2005	36148738	Histopathology	ICD-O C61	7.53	9.22	Pakzad^16^ (C)
Nation-wide	2006	36023018	Histopathology	ICD-O C61	7.82	9.57	Pakzad^16^ (D)
Nation-wide	2007	36166098	Histopathology	ICD-O C61	8.79	10.91	Pakzad^16^ (E)
Nation-wide	2008	35998071	Histopathology	ICD-O C61	10.37	12.8	Pakzad^16^ (F)
Nation-wide	2009	39346939	Histopathology	ICD-O	9.8	12.59	Basiri^17^
Nation-wide	2012	38420561	Histopathology	-	10.7	12.6	Almasi^9^
Semnan	1998-2002	37415	Histopathology	ICD-10	147	150	Babaei^18^
Tehran	1998-2001	15242718	Histopathology	ICD-O-2 & ICD-O-3	10.3	15.6	Mohagheghi^19^

NA, Not applicable; ASR, Age-standardized rate.
